# Knockout of Acinar Enriched microRNAs in Mice Promote Duct Formation But Not Pancreatic Cancer

**DOI:** 10.1038/s41598-019-47566-x

**Published:** 2019-07-31

**Authors:** Dhruvitkumar S. Sutaria, Jinmai Jiang, Ana Clara Azevedo-Pouly, Lais Wright, Julie A. Bray, Kristianna Fredenburg, Xiuli Liu, Jun Lu, Carolina Torres, Georgina Mancinelli, Paul J. Grippo, Vincenzo Coppola, Thomas D. Schmittgen

**Affiliations:** 1 Department of Pharmaceutics, College of Pharmacy, University of Florida, Gainesville, FL USA; 2 Department of Pathology, University of Florida, Gainesville, Florida USA; 3 National Center for Toxicological Research, Food and Drug Administration, Jefferson, AR USA; 4 Department of Cancer Biology and Genetics, College of Medicine and Comprehensive Cancer Center, Ohio State University, Columbus, Ohio USA; 5 Department of Pathology, Beijing Chaoyang Hospital, Capital University, Beijing, China; 6 Department of Medicine, University of Illinois, Chicago, Illinois USA

**Keywords:** Health sciences, Pancreatic cancer

## Abstract

The pancreatic acinar-enriched miR-216a, miR-216b and miR-217 are encoded within the miR217HG. These miRNAs have been purported to play a tumor suppressive role as their expression is reduced in both human and mouse pancreatic ductal adenocarcinoma (PDAC). To examine this possibility, we generated individual, germline knockout (KO) mice of miR-216a, miR-216b or miR-217. Unlike our previous study showing germline deletion of the miR217HG was embryonic lethal, CRISPR-Cas9 deleted portions of the 5’ seed region of the miRNAs produced live births. To investigate possible phenotypes during pancreatic acinar ductal metaplasia (ADM), pancreatic acini from wild type and KO mice were plated on collagen and allowed to transdifferentiate over 4 days. Acini from each of the three miRNA KO mice produced greater numbers of ducts compared to controls. Evaluation of the gene expression during *in vitro* ADM demonstrated an increase in Krt19 and a reduction in acinar genes (Carboxypeptidase A1, Amylase2a) on day 4 of the transdifferentiation. Recovery was delayed for the miR-216a and miR-216b KOs following caerulein-induced acute pancreatitis. Also predominate in the caerulein treated miR-216a and miR-216b KO mice was the presence of pancreatic duct glands (PDGs). To further establish a phenotype, miRNA KO mice were crossed with EL-*KRAS*^G12D^ (EK) mice and followed up to 13 months of age. While all mice developed severe dysplasia and cystic papillary neoplasms, there existed no apparent phenotypic difference in the miRNA KO/EK mice compared to EK mice. Our data does not support a tumor suppressor role for miR-216a, miR-216b or miR-217 in PDAC and emphasizes the need for phenotypic evaluation of miRNAs in complex *in vivo* models beyond that performed using cell culture.

## Introduction

Pancreatic ductal adenocarcinoma (PDAC) is the third most lethal cancer in the USA and is predicted to become the second most deadly cancer in the country by 2030^1,2^. It is widely understood that the cells of origin for PDAC are the exocrine acini^3–5^. Pancreatic acini display remarkable plasticity^6^ and exhibit a natural ability to dedifferentiate and then transdifferentiate into ductal-like cells following pancreatic injury by a reversible process known as acinar ductal metaplasia (ADM). The hallmarks of ADM include loss of acinar gene expression (e.g. amylase) and gain of ductal epithelial morphology and gene expression including cytokeratin 19 (KRT19)^7–9^. Pancreatic intraepithelial neoplasm (PanINs), one of the main precursor lesions to PDAC, arise from ADM^4^. ADM is the earliest known precursor lesion for PDAC making acinar to ductal transdifferentiation a key phase in the initiation of pancreatic cancer.

microRNAs (miRNAs) display altered patterns of expression in clinical specimens of pancreatic ductal adenocarcinoma (PDAC), with some miRNAs increased and others decreased in the tumors^10–12^. One particular miRNA cluster that has generated interest in PDAC are those miRNAs encoded within the miR217 host gene (miR217HG i.e. miR-216a, miR-216b and miR-217). miR217HG is a lncRNA that is located within chromosome 2p16.1 in humans and 11qA3.3 in mice. The processed, mature miR-216a, miR-216b and miR-217 are pancreas enriched^13,14^ with predominate, abundant expression in pancreatic acini^15^. miR-216a, miR-216b and miR-217 are also expressed in brain^16^ and to a lesser degree in kidneys, lung and small intestine^17^. miR-216a, miR-216b and miR-217 expression is reduced in mice and humans with pancreatitis^18–20^, during PanIN progression^21,22^ and in PDAC in both mice and humans^12,19,23,24^.

We and others posited that the miR-216/−217 cluster may be tumor suppressive in pancreas since other tissue specific/enriched miRNAs such as miR-122 in liver^25,26^ and miR-126 in muscle^27^ are tumor suppressive. Further evidence supporting a potential tumor suppressive role for these miRNAs in PDAC comes from an evaluation of their reported target genes that include *KRAS*^28,29^, *ANLN*^30^, *E2F3*^31^, *JAK2*^32^ and *TPT1*^33^. The oncogenic lncRNA *MALAT1* enhanced *KRAS* activity and PDAC development by sponging miR-217 and altering the miR-217 nucleus/cytoplasm ratio^34^. Validation of each of these miRNA/target gene interactions and their effects were obtained using cell culture systems and have not been replicated in transgenic mouse models to our knowledge.

In order to establish a functional role for the three mature miRNAs, we deleted a 27.9 kbp region of miR217HG in mice, however the deletion was embryonic lethal at around E9.5^23^. While the specific cause(s) of the lethality are unknown, possibilities include simultaneous deletion of all three miRNAs or deletion of a functional miR217HG lncRNA. To evaluate these possibilities in greater detail, we generated individual germ line KO mice for miR-216a, miR-216b and miR-217 using CRISPR/Cas9 gene editing. Portions of each mature miRNA including the 5′ seed regions were successfully deleted, and viable mice ensued. Phenotypic evaluation of the KOs, including effects on *in vitro* transdifferentiation, caerulein-induced acute pancreatitis and crossing KO mice with those harboring a mutant *KRAS*, produced subtle changes. The miRNA KO did not enhance metaplasia beyond advanced cystic papillary neoplasms (CPN), which was not different from control. We conclude that miR-216a, miR-216b and miR-217 are not tumor suppressive in pancreas and our work emphasizes the need to evaluate miRNA function beyond *in vitro* systems.

## Materials and Methods

All experimental methods were performed in accordance with the relevant guidelines and regulations.

### Generation of CRISPR/Cas9 miRNA knockout mice

All experiments conducted in this study were approved by the Institutional Animal Care and Use Committee (IACUC) at both the Ohio State University and the University of Florida. Single guide RNA sequences targeting miR-216a, miR-216b and miR-217 genes were designed using the CRISPR/Cas9 design tool (crispr.mit.edu, SFig. 1). Using the primer pairs for cloning (Supplemental Table 1), we annealed, phosphorylated and cloned the miRNA targeting sgRNA sequences within the px330 bicistronic expression plasmid (Addgene). PCR amplification was performed to add the T7 promoter sequence to the sgRNA template using primers 217 F and R, 216a-1 F and R, 216a-2 F and R, 216b-1 F and R and 216b-2 F and R (Supplemental Table 1). The T7-sgRNA PCR product was purified using Qiagen Gel purifying kit and served as a template for *in vitro* transcription using ampliscribe T-flash transcription kit (Epicentre, Madison, WI). The reaction was purified using Centri-Spin columns (Princeton separations, NJ) and sgRNAs were eluted in RNAse-free water and stored at −80 °C. Cas9 mRNA (Trilink Biotechnologies, San Diego, CA) was codon optimized, capped and polyadenylated mimicking the fully processed mature mRNA. Mutant mice were generated at the Genetically Engineered Mouse Modeling Core of the Ohio State University according to standard procedure^35,36^. Briefly, a mix of Cas9 mRNA (100 ng/uL, CleanCap™ Cas9 mRNA TriLink BioTechnologies) and miR-specific sgRNA (50 ng/uL) was injected into the cytoplasm of fertilized C57Bl/6N zygotes. Following micromanipulation, zygotes were transferred to pseudo-pregnant recipient mothers and allowed to develop to term. To mitigate off-targets effects, mutant mice were crossed into C57Bl/6 for 5 generation before use for experimental purposes. The mfold web server determined if the deletions produced any changes in the folding of the pre-miRNA sequences. Based on the mfold results and TOPO sequencing, the mutant pups that exhibited the largest length of deletions were selected for breeding to obtain a pure biallelic miRNA KO.

### High resolution melt analysis and Sanger sequencing

Genomic DNA isolated from the mouse’s tails that were born following the injection of the sgRNA and Cas9 mRNA was PCR amplified using primers (Supplemental Table 1) for 98 °C for 2 mins; 35 × (98 °C for 5 secs, 60 °C for 5 secs); 65–95 °C (in 0.5 °C increments) 5 sec/step. The PCR reaction was performed using Evagreen dye on a Biorad CFX96 PCR system. Several mutants were identified from the pool of pups that were obtained after CRISPR/Cas9 injections. PCR amplification was carried out on the positive mutants that were identified through high resolution melt analysis and were cloned into a TOPO vector using Original TA cloning kit (Invitrogen, USA). Sanger sequencing was performed to verify and determine the mutations that occurred in the miRNA transcript.

### qRT-PCR

RNA was isolated from the mouse pancreata using Trizol reagent^37^. All RNA samples had an RNA integrity number (RIN) of 7.5 or greater. cDNA was synthesized to the mature miR-216a, miR-216b and miR-217 as described^38^, 18S rRNA was used as the internal control and data were analyzed using the comparative C_T_ method. Situations did not exist where a significant change was observed in the 18S rRNA expression between test and control groups (fold change <1.5, P > 0.05). miRNA expression was considered not expressed if the mean C_T_ ≥ 36.

### Acinar-ductal transdifferentiation

Pancreatic acinar cell culture was performed as previously described by Shi *et al*., with several modifications^39^. Rat tail collagen (Invitrogen) was neutralized and 250 µl was added per well of a 24 well plate to provide a firm attachment base. Freshly isolated mouse pancreata were washed in cold HBSS and dissected into small pieces. Acini were digested with 5 ml of 0.2 mg/ml Collagenase P (Roche) and incubated at 37 °C for 30 mins. HBSS/5% FBS was added to inactivate the collagenase, isolated acini were washed three times with HBSS and filtered through a 100 µm mesh. Cell suspensions were layered onto HBSS/30% FBS and centrifuged at 180 x g for 3 mins. Pellets were resuspended in Waymouth’s media supplemented as described^39^. Acini were mixed 1:1 with neutralized collagen and 0.5 ml of this mixture was plated onto the collagen layer in the 24 well plate. Following 1 hr at 37 °C, 1 ml of culture media was added to each well. An eight mm^2^ adhesive grid (Sigma) was affixed to the bottom of each well and microscopic acini and ducts were manually counted. To isolate RNA from the cultures, collagen was digested in collagenase P solution at 37 °C for 30 mins with shaking. Acini were pelleted and total RNA was isolated using the RNAesy Kit (Qiagen). Random cDNA was prepared using Superscript II polymerase (Invitrogen) and qPCR was performed using SYBR green reagent detection. Primers used in the study are provided in Supplemental Table 1.

### Acute pancreatitis

Acute pancreatitis was induced in the mice using the cholecystokinin analogue caerulein, purchased as the ammonium salt (Bachem, Torrance, CA). miRNA KO and control C57/B6 mice were injected hourly with 8 injections of caerulein (100 µg/kg) on two consecutive days, for a total of 16 injections. Mice were euthanized at day 2, day 4 and day 7 following the final caerulein injection. Pancreas tissues were removed from the animals, fixed in formalin, embedded into paraffin, sectioned and stained with Hematoxylin and Eosin. For all of the caerulein treated and saline controls, serial sectioning was performed of the entire pancreas tissue and H&E staining was carried out on every fifth slide to confirm the presence or absence of PDGs. For semi-quantification, slides were analyzed by two different investigators in a blinded fashion. A total of 6 mice were used for each treatment arm (caerulein or saline). The entire slide section was evaluated. The percentage of ADM was estimated by the presence of ADM compared to the overall tissue. The degree of inflammation, fibrosis and atrophy was scored as +, ++, +++.

### Tissue histology analysis of miRNA-KO Kras crossed mice

All the mice during the study were bred and maintained at the University of Florida. Genetically engineered mice of the following genotypes were used: EL-KRAS^G12D^ (or EK), miR-216a/EK, miR-216b/EK and miR-217/EK. Where EL stands for Elastase promoter; Kras stands for activating Kras mutation (G12D), EK for EL-KRAS^G12D^; miR-216a, miR-216b and miR-217 have deletions on those microRNAs, respectively. Mice were euthanized at 3, 6 and 13 months of age. A minimum of 5 mice were used for each comparison. The pancreas was removed, fixed in 10% formalin, processed, sectioned at 4 µm, and stained with Hematoxylin and Eosin for histological evaluation. Periodic acid Schiff (PAS) staining was done to confirm the presence of mucins within the PDGs. To evaluate the differences in the pancreatic neoplastic phenotypes, tissue slides were scanned using Aperio Microscope and then blinded for evaluation by two different investigators using the Aperio Image Scope software (Leica, Wetzlar, Germany) which provides tools to measure and quantify selected areas. The whole area of the tissue was measured and used to normalize the frequency of lesions (total number of lesions, CPNs and cysts, per tissue area in mm^2^). The percentage of parenchyma replaced by fat (lipoatrophy) was also obtained by dividing total lipoatrophy areas (mm^2^) by the total tissue area (mm^2^) To estimate the percentage of normal tissue, ADM and fibrosis present in each sample, a 4x magnification of the tissue was used and five different fields of views were assessed, quantified and averaged. The area of the five largest lesions (mm^2^) was measured and averaged. Each group was compared with the age-matched EL-Kras control group. Possible outliers were identified using GraphPad and differences between the groups were assessed by One-Way ANOVA and only values p < 0.05 were considered significant. For comparing all groups and ages, Two-Way ANOVA was used for statistical purposes and only values p < 0.05 were considered significant.

## Results

### Development of miR-216/−217 knockout mice

The miRNAs encoded within the miR-217HG have been reported as enriched in pancreas^13–15^. The NIH Genotype-Tissue Expression (GTEx) project data set on 51 normal human tissues was mined to confirm these findings (SFig. 2). To evaluate a possible role for these miRNAs in maintaining acinar cell identity or tumor suppression, we generated individual germ line KOs of miR-216a, miR-216b and miR-217. While conditional gene deletion is the preferred method to study a tissue specific phenotype, we presumed that since the miR217HG is predominately expressed in pancreas (SFig. 2), germ line deletion would serve as a *de facto* conditional deletion in pancreas. CRISPR/Cas9 gene editing was used to delete a small portion of each mature miRNA that included the 5’ seed region. High resolution melt analysis showed that 2 out of 23 pups were positive for miR-217 mutations, 6 out of 48 mice pups were positive for miR-216a mutations and only one pup out of 15 pups was identified as a mutant for miR-216b targeting (SFig. 3A). TOPO cloning and Sanger sequencing verified the deletions that occurred in the miRNA transcripts (SFig. 3B).

### miRNA and mRNA expression in KO mice

**Figure 1:**
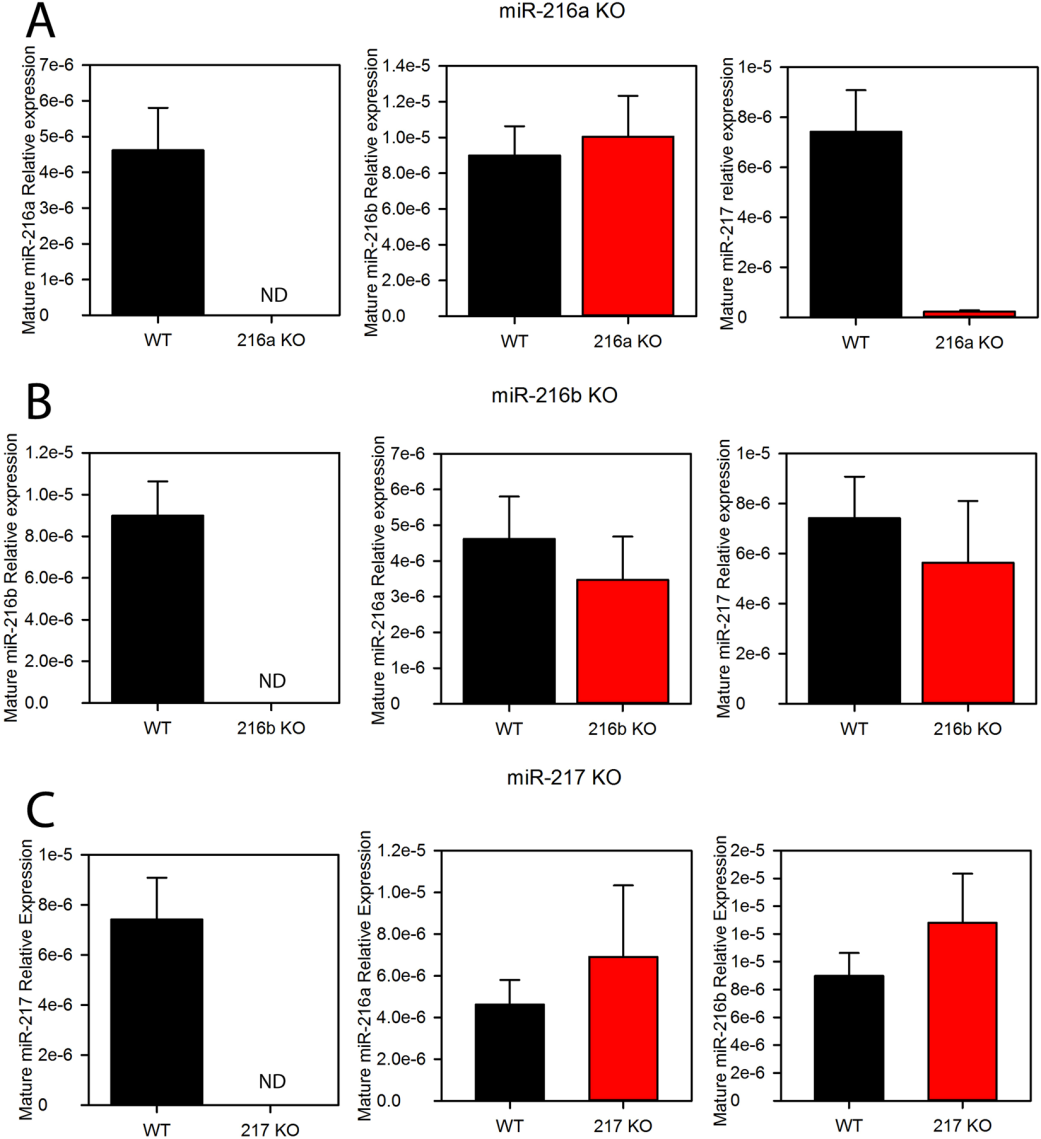
miRNA expression in the pancreata of individual knockout mice. TaqMan qRT-PCR was performed on miRNAs isolated from the pancreata of 13 month old A. miR-216a, B. miR-216b and C. miR-217 knockout mice. The expression of each individual miRNA, relative to 18S rRNA, is depicted on the y-axis of each graph. ND, not detectable.

Successful KO of each miRNA in mouse pancreata were confirmed by qPCR (Fig. 1). In mouse, miR-216a and miR-217 lie on the same pre-miRNA while miR-216b is on a separate transcript (SFig. 1). We wondered if deletion of one miRNA would alter the expression of another miRNA in the miR217HG cluster. In the miR-216a KO, miR-217 was also reduced by >95%. These phenomena existed in both young (1 month, not shown) and old (13 month, Fig. 1) KO mice. However, in both the miR-217 and miR-216b KOs, only the affected miRNA was reduced (Fig. 1). Since miR-216a lies upstream of miR-217, deletion of the upstream miRNA possibly affected processing of the downstream miRNA. We next performed an unbiased, gene expression profiling array that contained both coding and noncoding RNAs on RNA extracted from the pancreas of 6 week old miR-217 KO and wild type control mice. Unexpectedly, there was little significant change in gene expression from the cDNA array analysis of the miR-217 KO and wild type mice (SFig. 4). miR-217 was one of the few genes that were differentially expressed in the KO animals (fold change <1.5, P < 0.05). Three members of the Reg3 family (Reg3a, b and g) were increased in the miR-217 KO mice (SFig. 4).

### KO of miR-216a, miR-216b and miR-217 alters *in vitro* ADM

**Figure 2:**
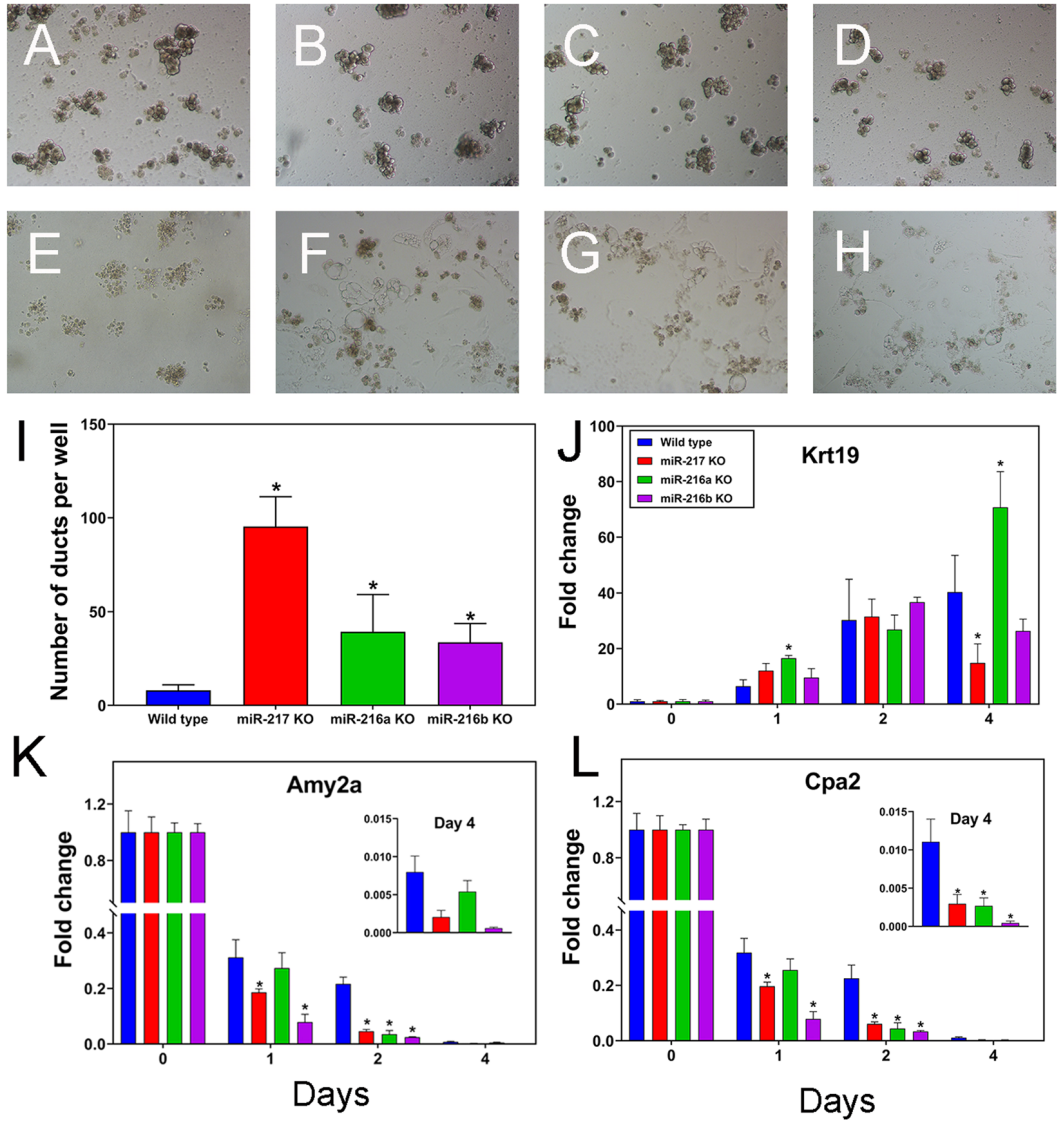
miRNA knockout accelerates *in vitro* pancreatic acinar ductal metaplasia. Mouse pancreatic acinar cells were plated onto collagen and the transdifferentiation occurred over 4 days. Bright field images were captured at Day 1 (**A**–**D**) and Day 4 (**E**–**H**) of culture for the wild type (**A**,**E**), miR-217 (**B**,**F**), miR-216a (**C**,**G**) and miR-216b (**D**,**H**) KOs. (**I**) Number of ducts after 4 days of culture in the wild type and individual knockout mice. RNA was isolated from the cultures at days 1 to 4 of the transdifferentiation and the expression of J. cytokeratin 19, K. amylase 2A and L. carboxypeptidase A2 was determined by qRT-PCR. Data are presented relative to 18S rRNA (mean ± SEM) and were normalized to the day 0 controls. *P < 0.05 (Student’s t-test).

In transgenic mouse models of PDAC, neoplasia formation is proceeded by the transdifferentiation of acinar cells into cells that display ductal epithelial gene expression and morphology (i.e. acinar ductal metaplasia or ADM). Changes in gene expression include reduction in acinar (Amy2a, Cpa2) and increased expression of ductal epithelial genes such as Krt19. ADM was studied experimentally by culturing the pancreatic acini on collagen from each of the three miRNA KO mice as well as control mice. When cultured on collagen in the absence of additional growth factors such as TGFα, ADM should be greatly reduced in wild type mice. Since miR-216a, miR-216b and miR-217 are highly enriched in pancreatic acini, we hypothesized that ADM would be enhanced in the KOs compared to wild type mice. As anticipated, the number of ducts increased for the three KO mice compared to wild type control (Fig. 2A–I). To further explore the effects of the miRNAs on ADM we quantified the expression in both acinar and ductal genes by qPCR. In the wild type mice, acinar genes decreased while Krt19 increased over the 4 day ADM experiment (Fig. 2J–L). While the trends were not consistent, there was a tendency for the acinar (Amy2a, Cpa2, Cpb1 and Ela1) gene expression to be reduced while Krt19 and Krt7 expression increased in the miRNA KOs over the 4 day ADM period (Fig. 2J–L, SFig. 5).

### Acute pancreatitis is enhanced in miRNA KO mice

**Figure 3:**
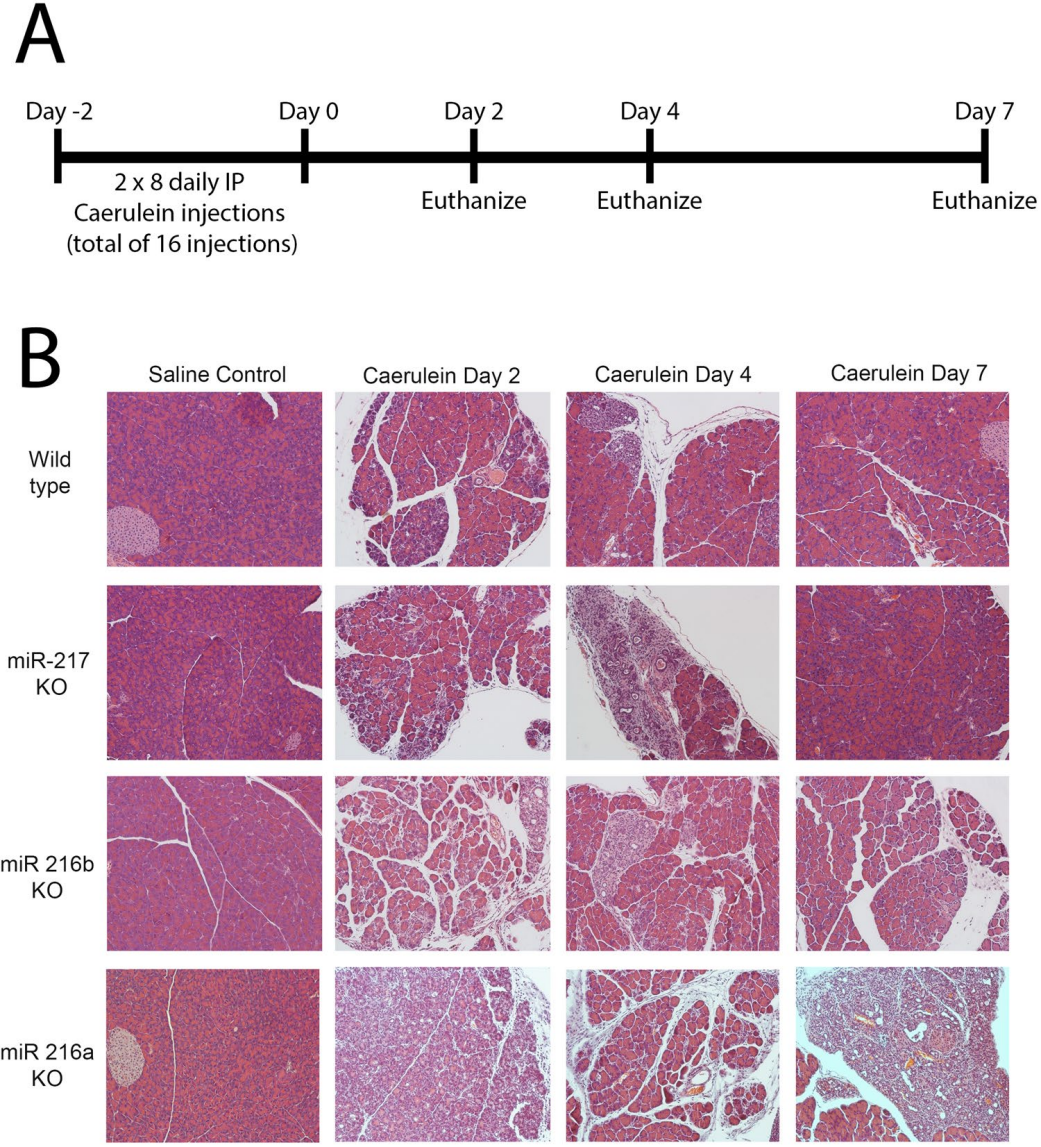
Recovery from acute pancreatitis. A. Wild type or miR-217, miR-216b or miR-216a knockout mice (2 month old) were dosed daily with eight injections of caerulein for two days. B. Mice were sacrificed at 2, 4 and 7 days following the last caerulein dose and the pancreata were processed for histology. 20X magnification.

Acute pancreatitis was induced following 16 intraperitoneal injections of caerulein in the wild type control and 3 miRNA KO mice (Fig. 3A). Pancreata were harvested at three different time-points and H&E staining was performed to evaluate histological changes (Fig. 3B). Caerulein-induced acute pancreatitis is a reversible phenomenon and complete acinar regeneration occurs by day 7. Caerulein induced extensive acinar injury, atrophy and increased inflammation in all the three KOs compared to WT mice (Fig. 3B, Supplemental Table 2). miRNA KO mice also displayed an increase in acinar cells undergoing ADM (Supplemental Table 2). The three KO mice exhibited increased ductal formation which was highly evident at day 2 in miR-216a and 216b KO and day 4 in miR-217 KO (Fig. 3B). By day 7, pancreata of the miR-217 and miR-216b KOs completely regenerated, however the pancreas of miR-216a KO did not (Fig. 3B).

**Figure 4:**
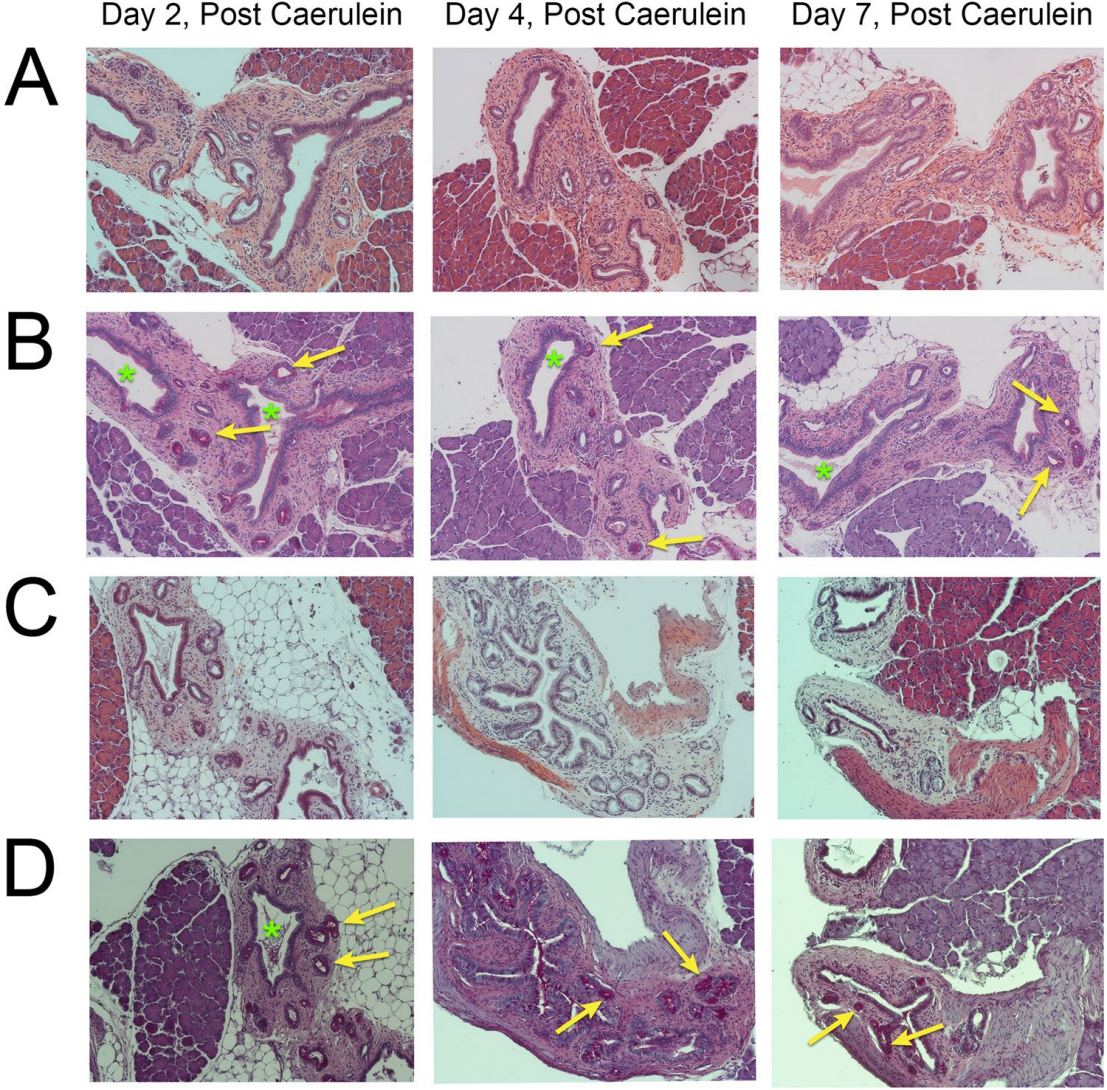
Presence of pancreatic ductal glands during acute pancreatitis. The presence of pancreatic ductal glands (PDGs, yellow arrows) was observed in the miR-216b and miR-216a knockout mice 2, 4 and 7 days, following caerulein-induced acute pancreatitis. Pancreata were stained with A,C hematoxylin and eosin or B,D periodic acid Schiff (PAS). Green asterisks represent enlarged ducts on the PAS stained sections. 20X magnification.

Pancreatic ductal glands (PDGs) are a distinct compartment that have been associated with chronic pancreatic injury and may be linked to PDAC development through the formation of intraductal papillary mucinous neoplasms (IPMNs)^40^. While PDGs were present in one of the caerulein treated wild type mice at day 4 (not shown), caerulein increased the PDGs formed in several of the miR-216a and miR-216b KOs (Fig. 4). PDGs were confirmed in the mouse pancreata for the presence of mucins by positive PAS staining (Fig. 4B,D). While the miR-216b KO exhibited PDGs at all three time points, we did not observe PDG structures in vehicle controls for miR-216a KO. The PDG structures observed on day 4 in miR-216a KO mice appeared to be undergoing hyperplasia.

### Lack of consistent phenotype when miRNA KO mice are crossed with EK mice

**Figure 5:**
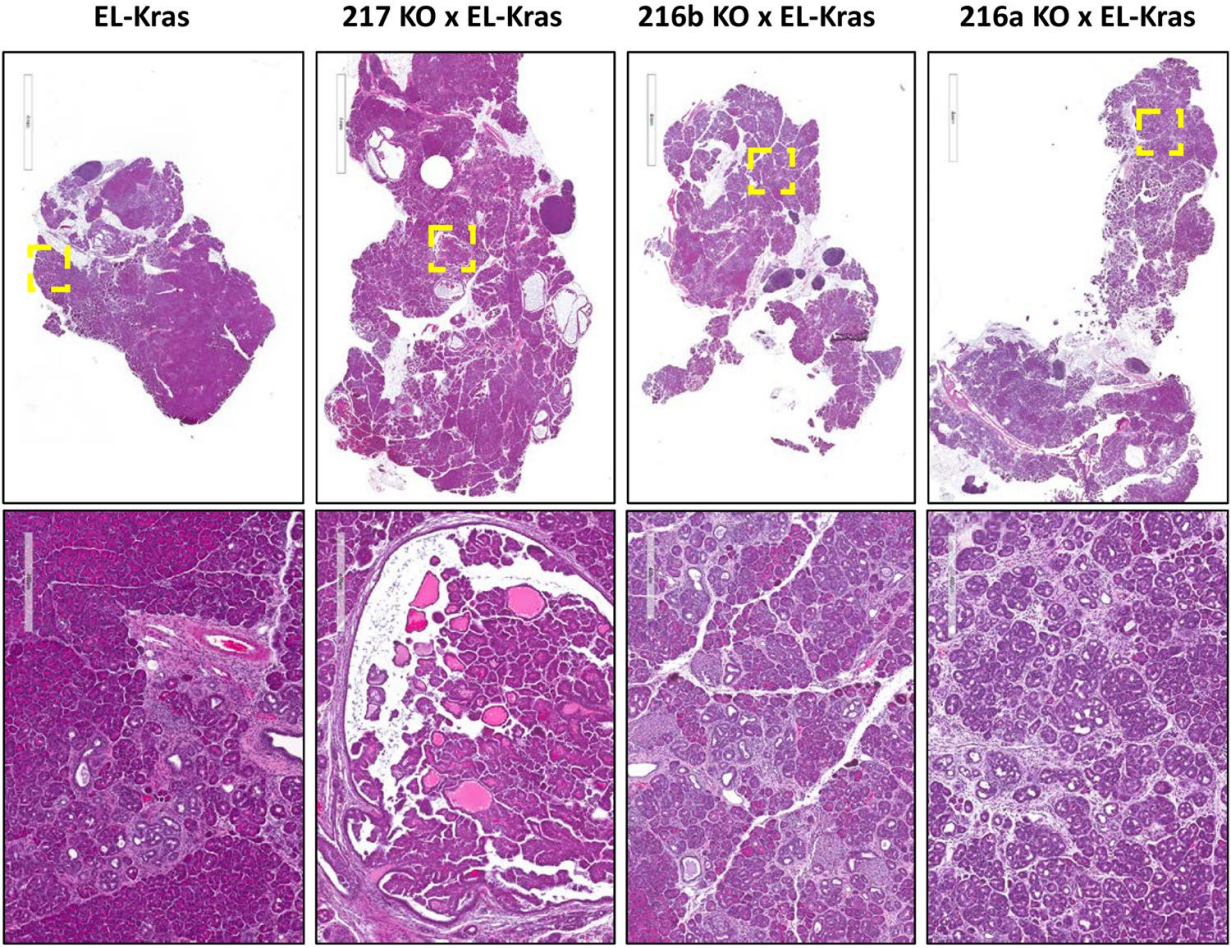
Histopathology of miRNA knockout and *Kras*^*G12D*^ mouse crosses. The miR-216a, miR-216b and miR-217 KO mice were crossed with the EL-*KRAS*^G12D^ mice. The histopathology from the pancreata of 3 month old mice are shown at 4× (upper panels) and 20× (lower panels) magnification.

We next questioned if a phenotype may be enhanced when the miRNA KO mice were bred to mice harboring an activating Kras mutation. EK mice were used as these mice have been shown to develop CPNs by 2–3 months of age that progressively become larger though only infrequently more severe^41^. miR-216a/EK, miR-216b/EK and miR-217/EK mice as well as EK controls were sacrificed at 3, 6, and 13 months of age. Upon histological evaluation, widespread dysplasia was apparent in the EK miRNA KO crosses by 3 months of age (Fig. 5). Dysplasia was also observed in the 6 and 13 month old mice (SFigs 7 and 8). A variety of histopathological lesions were quantified (e.g. total lesions, total lesion area, % normal tissue, % fibrosis, % ADM, % lipoatrophy and lesion size) in a blinded fashion on all three age groups of mice, however none of these differed between the KC and miR-216a/EK, miR-216b/EK or miR-217/EK mice with the exception of one lipoatrophy comparison (SFig. 9, Supplemental Table 3).

## Discussion

Many studies have identified differential miRNA expression in the tumors of patients with PDAC [reviewed in^42^]. Studies using cancer cell lines describe relationships between differential miRNA expression and alterations in cell proliferation, colony formation, induction of apoptosis and migration/invasion, suggesting that miRNAs may play a tumor suppressive or promotion role in PDAC^28,34,43–45^. More definitive evidence for such a role is generally gathered following gene knockin or knockout experiments in mice. We are aware of only one miRNA KO mouse that was developed in the context of pancreatic cancer, miR-324, which produced no phenotype towards the development of pancreatic acinar carcinoma^46^.

For simplicity, we generated germline miRNA KO mice rather than conditional KOs as the miR217HG cluster is predominately expressed in pancreas (SFig. 2) and^13,14^. One disadvantage of our approach is that compared to conditional KOs engineered to be inducible at the adult stage, the deleted miRNA gene may undergo compensation at the germline level. Compensation occurred between miR-34b/c and miR-449; miRNAs that are present on different chromosomes but share the identical 5′ seed sequence^47^. Evidence to support gene compensation in our study was mixed. Germline deletion of the highly pancreas-enriched miR-217 produced very little differential gene expression in the pancreas (SFig. 4), so if compensation was present, it was not evident from the gene expression profiling data. qPCR analysis of the other two miRNAs in the cluster that were not deleted only showed differential expression for miR-217 in the miR-216a KO, however its expression was reduced and not increased (Fig. 1). Of interest is that three members of the Reg3 family (Reg3a, b and g) were increased in the pancreata of the miR-217 KO mice (SFig. 4). Expression of the REG3 family are enriched in the pancreas and terminal ileum. Reg3g promoted PDAC in mice^48,49^ and REG3A/G were highly expressed in the ADM lesions of PDAC patients^50^.

Our data suggest some acceleration of ADM in the miRNA KO mice in both cell cultures and in caerulein treated animals. While we are unsure of a specific mechanism to describe this phenomenon, increased expression of Reg3a, b and g is a possibility, at least for the miR-217 KO mice (SFig. 4). Addition of Reg3a protein to acinar cultures promoted duct formation through activation of the MAPK pathway^50^. We do not believe that miR-217 directly targets the 3′ UTR of the Reg proteins since these interactions are not predicted using miRNA target algorithms such as TargetScan, though indirect regulation is possible.

While some of the older mice in our study developed neoplasia, we failed to demonstrate a pronounced and consistent phenotype in mice even among the 13 month-old miRNA KO/EK crosses. So, our findings paralleled those of Morris, *et al*., who deleted the miRNA processing enzyme Dicer in the pancreas using a Pdx1 driven Cre strain^51^. They report that Dicer knockout resulted in *Kras* associated ADM however Dicer loss did not accelerate *Kras* driven PDAC. It is possible that while KO of the miRNAs promote ADM and some PanIN lesions, it does not promote PDAC. Another explanation for a lack of phenotype in our miRNA KO/EK crosses is that the *Kras* mini gene used in developing the EK mice uses the metallothionein 3′UTR rather than the *Kras* 3′UTR^41^. As both miR-217^29^ and miR-216b^28^ bound to the 3′UTR of *Kras* and regulated Kras levels *in vitro*, any potential regulation of mutant *Kras* by these miRNAs would be lost in our miRNA KO/EK mice. In conclusion, our findings highlight the complexity associated with assigning tumor-promoting phenotypes with miRNAs and emphasize the need for studying these molecular events using *in vivo* models of cancer.

## Supplementary information

Supplemental Dataset 1

Supplemental Table 2

Supplemental Table 3
