# Supplementary material for: Knockout of Acinar Enriched microRNAs in Mice Promote Duct Formation But Not Pancreatic Cancer
Source: Sci Rep. 2019 Jul 31;9:11147. doi: 10.1038/s41598-019-47566-x (PMC6668398; doi:10.1038/s41598-019-47566-x)
Supplement: Supplementary file 1 — Supplemental Dataset 1 [file 41598_2019_47566_MOESM1_ESM.pdf]

Supplemental information for manuscript

Knockout of acinar enriched microRNAs in mice promote duct formation but not pancreatic cancer

Dhruvitkumar S. Sutaria[1], Jinmai Jiang[1], Ana Clara Azevedo-Pouly[3], Lais Wright[1], Julie A. Bray[2], Kristianna Fredenburg[2], Xiuli Liu[2], Jun Lu[5], Carolina Torres[6], Georgina Mancinelli[6], Paul J. Grippo[6], Vincenzo Coppola[4] and Thomas D. Schmittgen[1,*]

[1]College of Pharmacy and [2]Department of Pathology, University of Florida, Gainesville, Florida; [3]College of Pharmacy and [4]Department of Cancer Biology and Genetics, College of Medicine and Comprehensive Cancer Center, Ohio State University, Columbus, Ohio; [5]Department of Pathology, Beijing Chaoyang Hospital, Capital University, Beijing, China; [6]Department of Medicine, University of Illinois, Chicago, Illinois.

Supplemental Figure 1

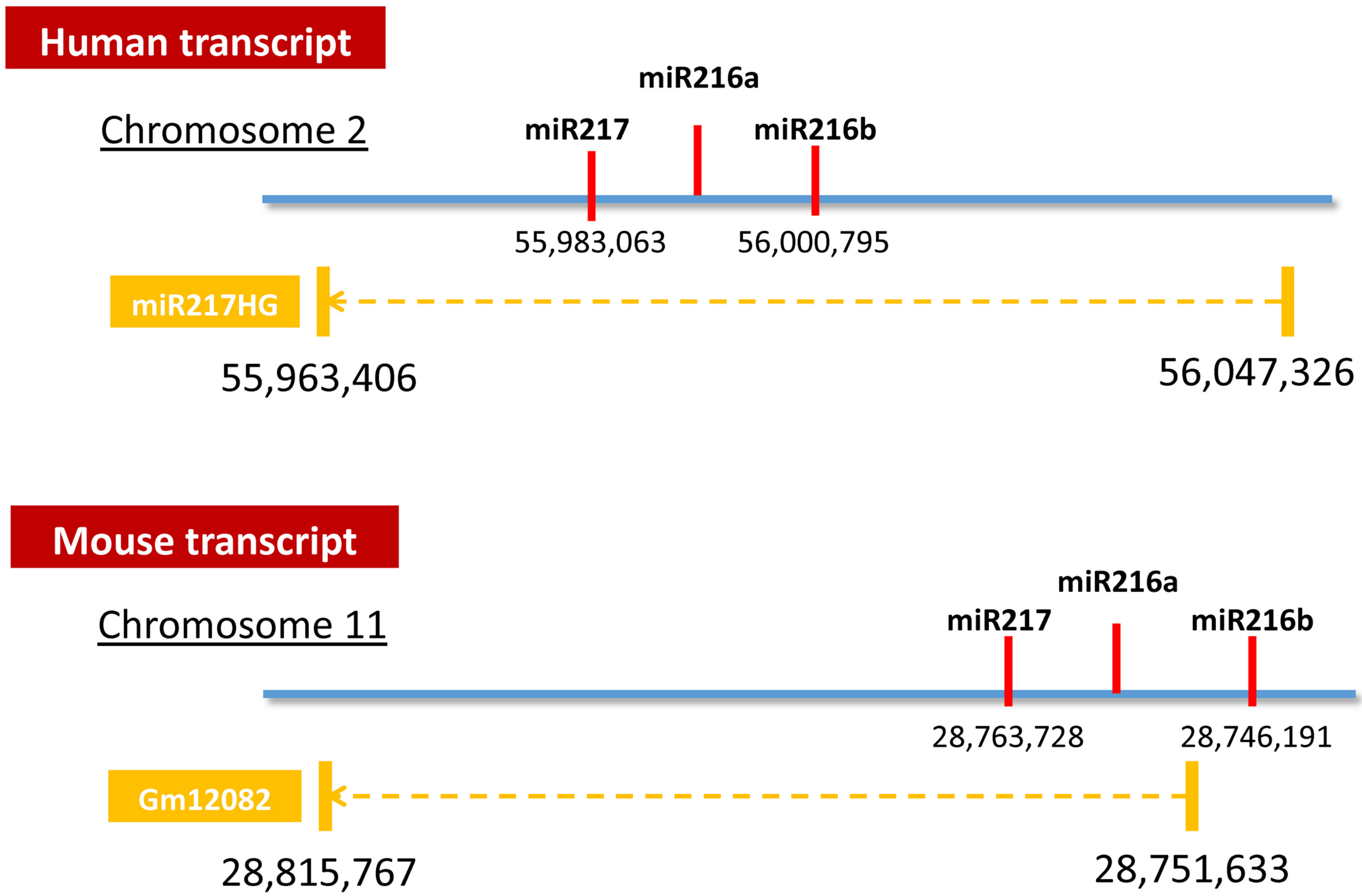

Supplemental Figure 1. Genomic maps of miR-217HG. The genomic location of the individual miRNAs located within miR217HG of A. human and B. mouse are shown.

Supplemental Figure 2

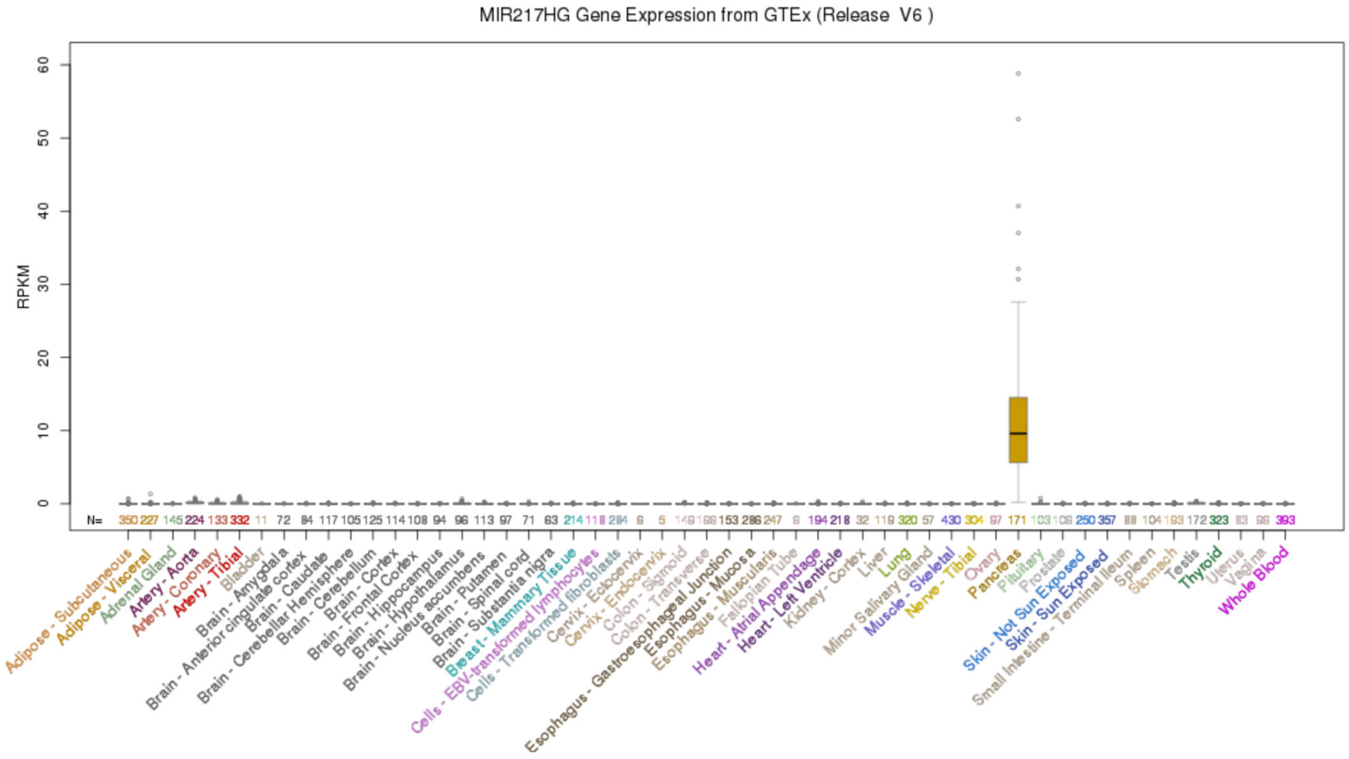

Supplemental Figure 2. miR217HG expression in human tissues. The NIH Genotype-Tissue Expression (GTEx) project was mined to examine the miR217HG expression levels in 51 tissues and 2 cell lines. Data are based on RNA-seq from the GTEx midpoint milestone data release (V6, October 2015). This release is based on data from 8555 tissue samples obtained from 570 adult post-mortem individuals.

Supplemental Figure 3

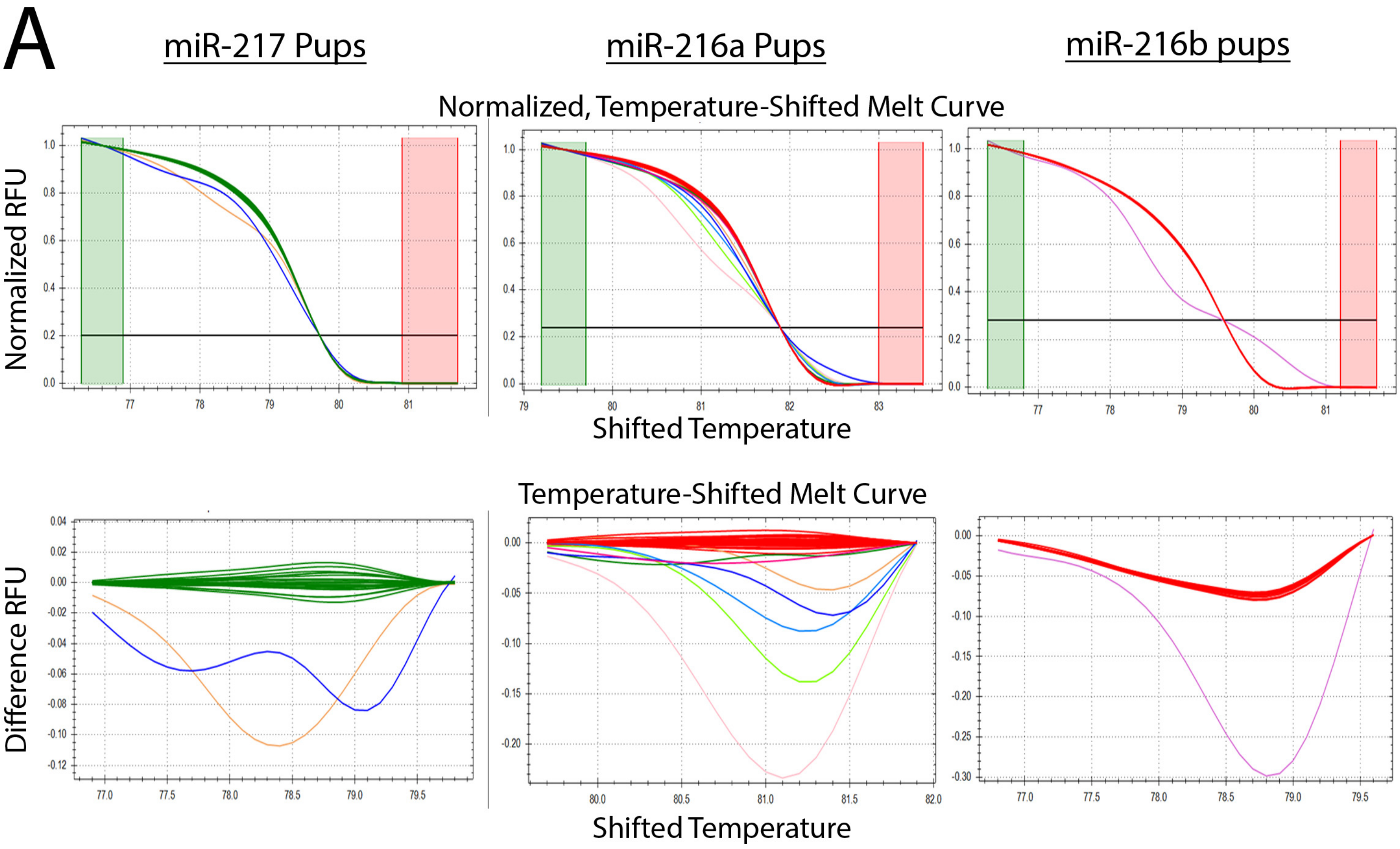

B

GTTTTTGATGTTGCAGATACTGCATCAGGAACTGACTGGATAAGACTTAATC WT-217
GTTTTTGATG…………………………………………………GATAAGACTTAATC -28 bp
GTTTTTGATGTTGCAG…………………………………………ACTTAATC -28 bp

TGAGTTGGTTTAATCTCAGCTGGCAACTGTGAGATGTCCCTATCATTCCTCACAGTGGTCTCTGGGATTA WT-216a
TGAGTTGGTTTAATCTCAG……………………………………………………TGGTCTCTGGGATTA -35 bp
TGAGTTGGTTTAATCTC……GGCAACTGTGAGATGTCCCTATCAT………………GTCTCTGGGATTA -14 bp

GCAGACTGGGAAATCTCTGCAGGCAAATGTGATGTCACTGAAGAAACCACACACTTACCTGTAGAGATTCTTC WT-216b
GCAGACTGGGAAATCT…………………………………………………………………TC -55 bp
GCAGACTGGGAAATCTCTGCAGGCAAATGTGATGTCACTGAAGAAACCACACACTTACCTGTAGAGATTCTTC -0 bp

Supplemental Figure 3. High resolution melt analysis and sequencing of clones. A. Mutants generated by CRISPR/Cas9 gene editing were identified by high resolution melt analysis following embryonic injection of the sgRNAs (red) for miR-217, 216a and 216b. B. Topo cloning followed by DNA sequencing confirmed the deletions generated by the CRISPR/Cas9 gene editing. Green, PAM sequence, Red, sgRNA binding sites.

Supplemental Figure 4

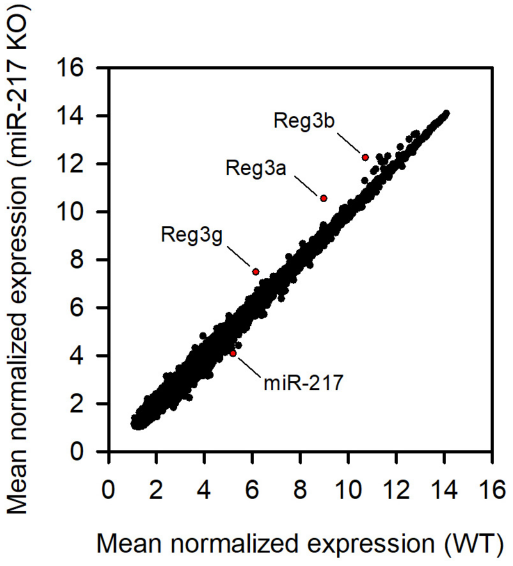

Supplemental Figure 4. Gene expression profile in pancreata from miR-217 KO and wild type mice. RNA isolated from the pancreas of three miR-217 KO and three wild type mice was assayed on a 55K gene expression array. Included on the array are both coding genes and noncoding RNAs. Data shown are for those 39K genes with known identifiers. The expression of miR-217 (fold change = -2.1, $P<0.05$) and Reg3a, b and g (fold change > 2.5, $P > 0.05$) are highlighted in red.

Supplemental Figure 5

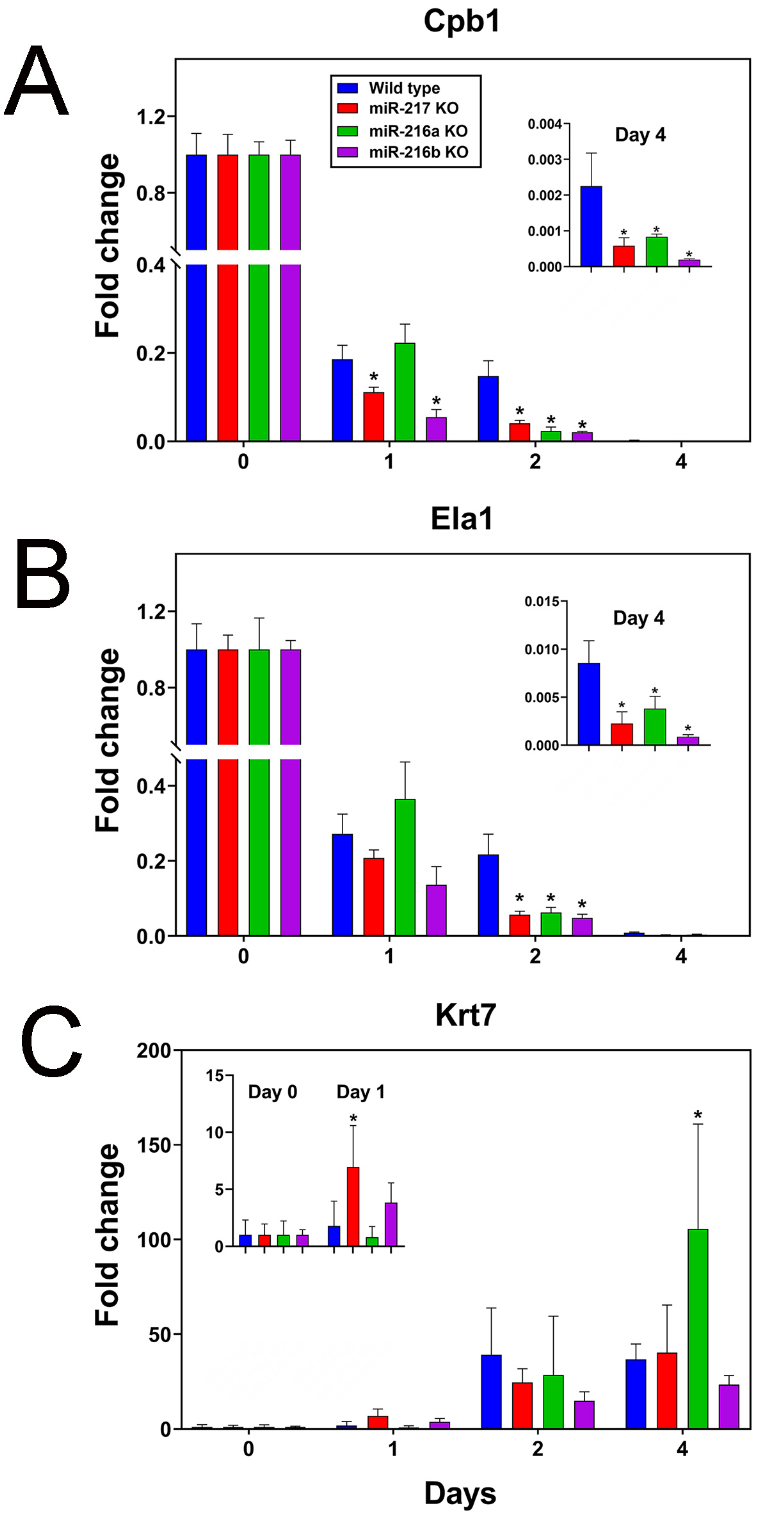

Supplemental Figure 5. In vitro ADM on pancreatic acini isolated from each knockout mouse. Pancreatic acinar cells were plated onto collagen and the transdifferentiation was quantified over a 4 day period. RNA was isolated from the cultures at days 1 to 4 of the transdifferentiation and the expression of A. carboxypeptidase B1, B. elastase 1 and C. cytokeratin 7 was determined by qRT-PCR. Data are presented relative to 18S rRNA (mean ± SEM) and were normalized to the day 0 controls. *, $P < 0.05$ (Student's t-test).

Supplemental Figure 6

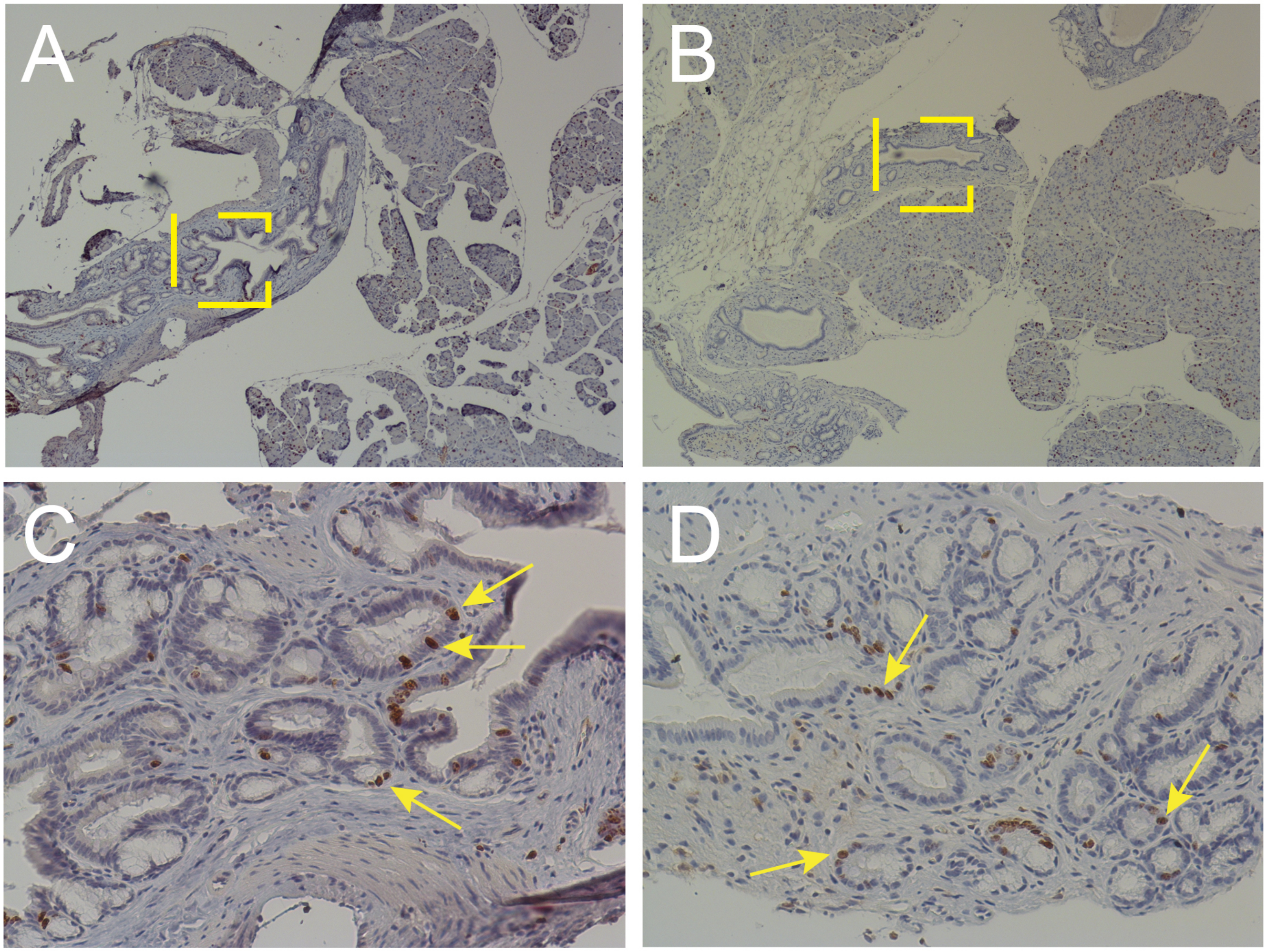

Supplemental Figure 6. PDGs in miR-216a KO mice exhibits proliferative activity. Paraffin sections from two different miR-216a KO mice (A,C) mouse 1 and (B,D) mouse 2 were immunostained for Ki67 and counter stained with hematoxylin and eosin. Arrow, positive staining for Ki67. (A,B) 4X and (C,D), 20X.

Supplemental Figure 7

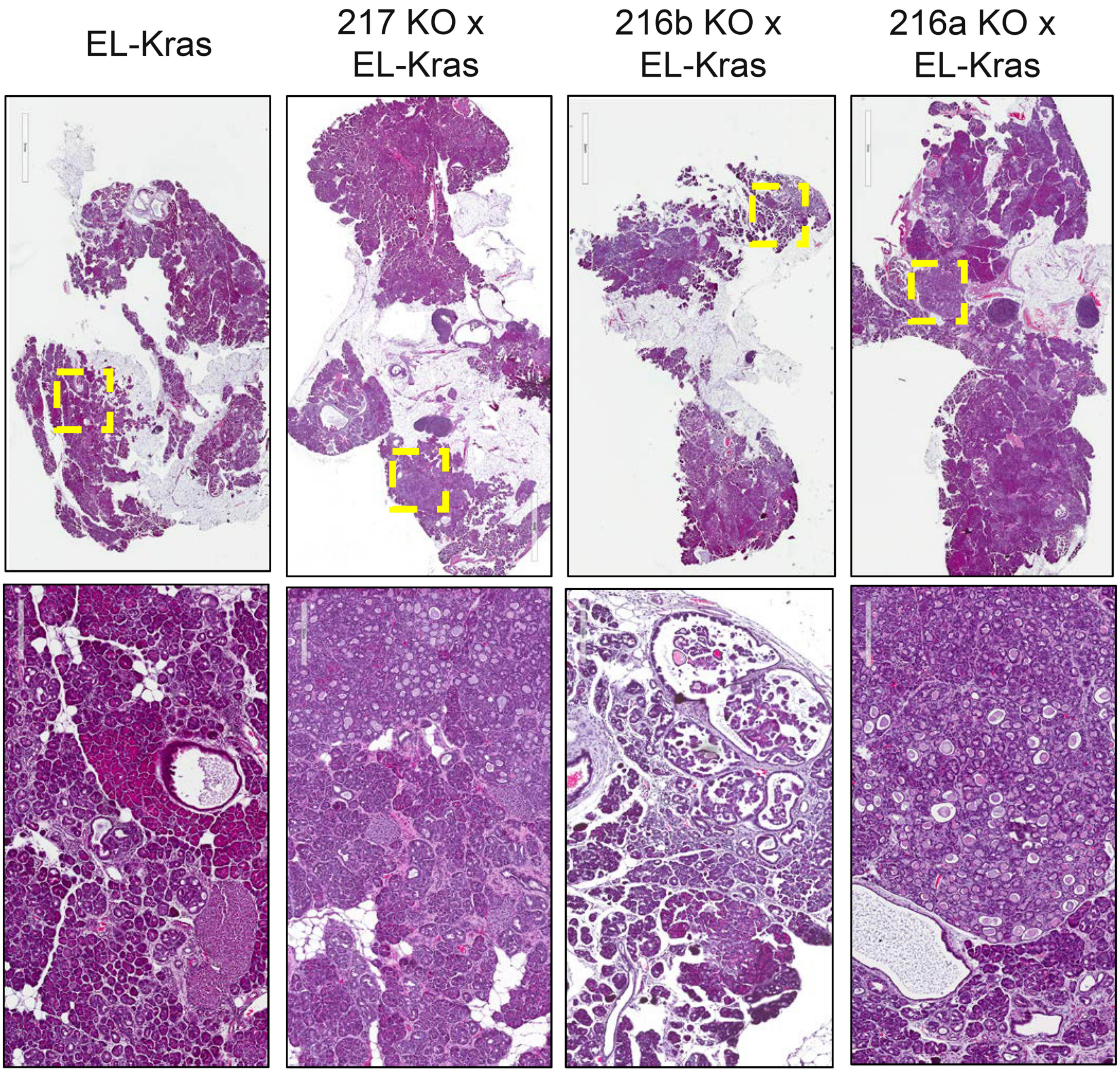

Supplemental Figure 7. Histopathology of miRNA knockout and KrasG12D mouse crosses. The miR-216a, miR-216b and miR-217 KO mice were crossed with the EL-KRASG12D mice. The histopathology from the pancreata of 6 month old mice are shown at 4× (upper panels) and 20× (lower panels) magnification.

Supplemental Figure 8

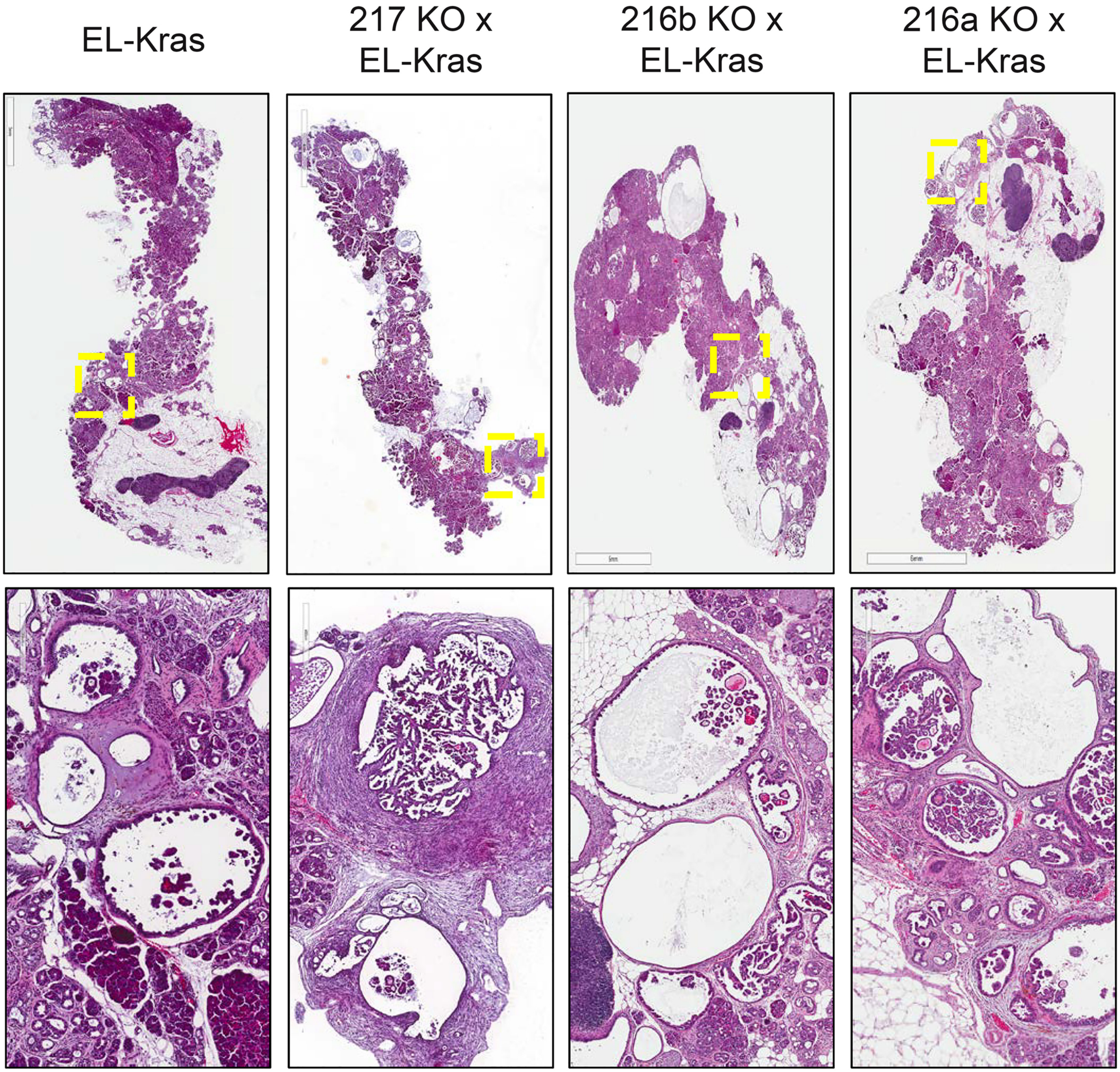

Supplemental Figure 8. Histopathology of miRNA knockout and KrasG12D mouse crosses. The miR-216a, miR-216b and miR-217 KO mice were crossed with the EL-KRASG12D mice. The histopathology from the pancreata of 13 month old mice are shown at 4× (upper panels) and 20× (lower panels) magnification.

Supplemental Figure 9

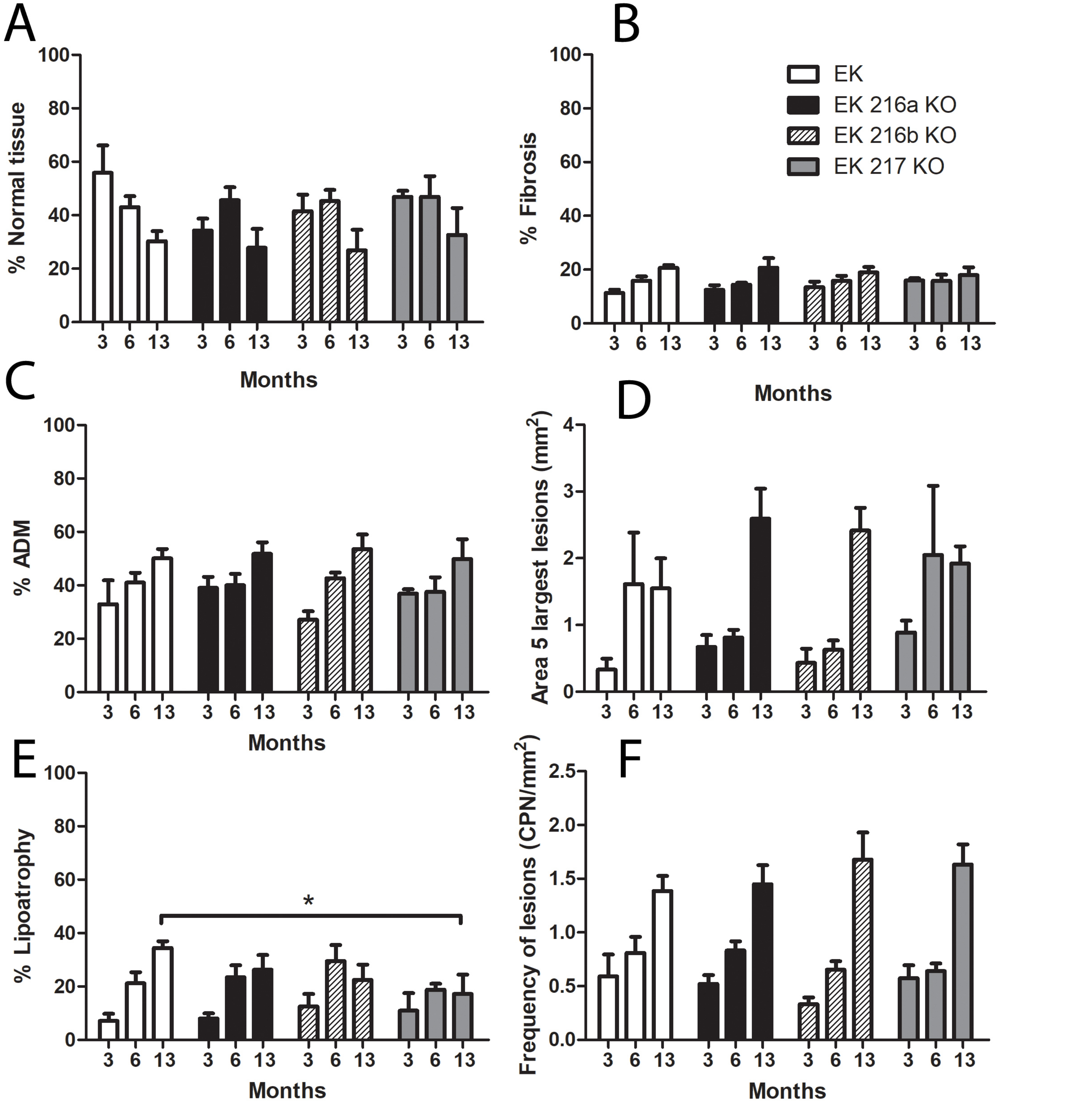

Supplemental Figure 9. Quantitative histopathology on miRNA knockout and KrasG12D mouse crosses. The miR-216a, miR-216b and miR-217 KO mice were crossed with the EL-KRASG12D mice. Mice of 3, 6 and 13 months old were sacrificed and their pancreata was observed histopathologically in a blinded fashion by two different investigators. The percentage of A. normal tissue, B. fibrosis, C. ADM, as well and D. the area of the 5 largest lesions, E. % lipoatrophy and F. frequency of lesions are reported. *, $P < 0.05$ (2-way ANOVA).

Supplemental Table 1. Primer and sgRNA sequences

No	Primer	Direction	Sequence (5'-3')
1	217 sgRNA	F	CACCGATGTTGCAGATACTGCATC
2		R	AAACGATGCAGTATCTGCAACATC
3	216a-1 sgRNA	F	CACCGTGAGTTGGTTTAATCTCAGC
4		R	AAACGCTGAGATTAAACCAACTCAC
5	216a-2 sgRNA	F	CACCGTAATCCCAGAGACCACTGTG
6		R	AAACCACAGTGGTCTCTGGGATTAC
7	216b-1 sgRNA	F	CACCGCAGACTGGGAAATCTCTGC
8		R	AAACGCAGAGATTTCCCAGTCTGC
9	216b-2 sgRNA	F	CACCGAATCTCTACAGGTAAGTGTG
10		R	AAACCACACTTACCTGTAGAGATTC
11	217 T7 transcription	F	TTAATACGACTCACTATAGATGTTGCAGATACTGCATC
12		R	AAAAGCACCGACTCGGTGCC
13	216a-1 T7 transcription	F	TTAATACGACTCACTATAGGTGAGTTGGTTTAATCTCAGC
14		R	AAAAGCACCGACTCGGTGCC
15	216a-2 T7 transcription	F	TTAATACGACTCACTATAGGTAATCCCAGAGACCACTGTG
16		R	AAAAGCACCGACTCGGTGCC
17	216b-1 T7 transcription	F	TTAATACGACTCACTATAGGCAGACTGGGAAATCTCTGC
18		R	AAAAGCACCGACTCGGTGCC
19	216b-2 T7 transcription	F	TTAATACGACTCACTATAGGAATCTCTACAGGTAAGTGTG
20		R	AAAAGCACCGACTCGGTGCC
21	217 HRM analysis	F	GCGTTGCTGTGTTGGGAAATA
22		R	GGTGTTTGCTGGTGGTTGTTT
23	216a HRM analysis	F	CAGTTCTACCTAGAGCCTCCAAGCA
24		R	GTTCTGATTCAGGTTGTCATGATCATC
25	216b HRM analysis	F	GCAAGACTAGAAAGACCATTGTAGAGT
26		R	GACAAGCCTTCATTCGGTTATTTC
27	Amylase	F	TTGCCAAGGAATGTGAGCGAT
28		R	CCAAGGTCTTGATGGGTTATGAA
29	Carboxypeptidase A2	F	GATCAAGAGCGTGAAGAGATGC
30		R	AGCCACGAGGTTATCCATTTCT
31	Cytokeratin 19	F	CCTCCCGAGATTACAACCACT
32		R	GGCGAGCATTGTCAATCTGT
33	Elastase	F	CGTGGTTGCAGGCTATGACAT
34		R	TTGTTAGCCAGGATGGTTCCC
35	Carboxypeptidase B1	F	AGGCATGGATTCAACAAGTTGC
36		R	AGCCTCTCTCACAAACCACTG
37	Cytokeratin 7	F	CACGAACAAGGTGGAGTTGGA
38		R	TGTCTGAGATCTGCGACTGCA
